# 

**Published:** 1958-07

**Authors:** 


					1/ >
\f?\
THE
MEDICAL JOURNAL
OF THE
SOUTH-WEST
Incorporating
The Bristol Medico-Chirurgical Journal
July 1958
VOL. 73 (iii). No. 269 Price 4./-

				

